# Clinical utility of fungal culture and antifungal susceptibility in cats and dogs with histoplasmosis

**DOI:** 10.1111/jvim.16725

**Published:** 2023-04-24

**Authors:** Andrew S. Hanzlicek, Kate S. KuKanich, Audrey K. Cook, Steven Hodges, John M. Thomason, Rupika DeSilva, Akhilesh Ramachandran, Michelle M. Durkin

**Affiliations:** ^1^ Department of Veterinary Clinical Sciences, College of Veterinary Medicine Oklahoma State University Stillwater Oklahoma USA; ^2^ MiraVista Diagnostics Indianapolis Indiana USA; ^3^ Department of Clinical Sciences, College of Veterinary Medicine Kansas State University Manhattan Kansas USA; ^4^ Department of Small Animal Clinical Sciences, College of Veterinary Medicine and Biomedical Sciences Texas A&M University College Station Texas USA; ^5^ Oklahoma Veterinary Specialists Tulsa Oklahoma USA; ^6^ Department of Clinical Sciences, College of Veterinary Medicine Mississippi State University Mississippi State Mississippi USA; ^7^ Oklahoma Animal Disease Diagnostic Laboratory, Department of Veterinary Pathobiology, College of Veterinary Medicine Oklahoma State University Stillwater Oklahoma USA

**Keywords:** canine, feline, fungal infection, histoplasma, invasive fungal infection

## Abstract

**Background:**

Culture can be used for diagnosis and antifungal susceptibility testing in animals with fungal infections. Limited information is available regarding the diagnostic performance of culture and the susceptibility patterns of *Histoplasma* spp. isolates.

**Hypothesis/Objectives:**

Describe the clinical utility of culture and the susceptibility patterns of *Histoplasma* spp. isolates causing histoplasmosis in cats and dogs.

**Animals:**

Seventy‐one client‐owned animals, including 33 cats and 19 dogs with proven or probable histoplasmosis.

**Methods:**

Culture was attempted from tissue or fluid samples. Diagnostic performance of culture, cytopathology, and antigen detection were compared with final diagnosis. Susceptibility to antifungal agents was determined for a subset (11 from dogs, 9 from cats) of culture isolates.

**Results:**

Culture had a diagnostic sensitivity of 17/33 (52%; 95% confidence interval [CI], 34%‐69%) and 15/19 (79%; 95% CI, 61%‐97%) and specificity of 6/6 (100%; 95% CI, 54%‐100%) and 10/10 (100%; 95% CI, 69%‐100%) in cats and dogs, respectively. Culture was not positive in any animal in which cytopathology and antigen testing were negative. Target drug exposure (area under the concentration curve [AUC]/minimum inhibitory concentration [MIC] >25) should be easily achieved for all isolates for itraconazole, voriconazole, or posaconazole. Five of 20 (25%) isolates had fluconazole MIC ≥32 μg/mL and achieving target drug exposure is unlikely.

**Conclusions and Clinical Importance:**

Fungal culture did not improve diagnostic sensitivity when used with cytopathology and antigen detection. Susceptibility testing might help identify isolates for which fluconazole is less likely to be effective.

**Abbreviations**
AUC: area under the curve
EIA: enzyme immunoassay
ID: agar gel immunodiffusion
MIC: minimum inhibitory concentration

## 1 INTRODUCTION

Histoplasmosis, caused by the dimorphic fungus *Histoplasma* spp., is an enzootic invasive fungal infection affecting mammals worldwide. In North America, most infections are found east of the Rocky Mountains and in central California. Infection is by inhalation of microconidia found in the environment, and development of disease reflects the interactions of inoculum size, host immunity, and fungal virulence.^1^ Disease can be localized to the respiratory tract or disseminate to any organ in the body.^2^, ^3^, ^4^ After dissemination, disease often is multisystemic but can be localized.^2^, ^3^, ^4^ Domestic cats are more commonly affected as compared to domestic dogs, but histoplasmosis is an important clinical consideration in both species.

Clinical signs are variable and often nonspecific, overlapping with other infectious or inflammatory diseases.^2^, ^5^ Diagnosis often is delayed, with an average duration of clinical signs of 6 and 4 weeks in cats and dogs, respectively.^2^, ^5^ Identification of *Histoplasma* spp. organisms within tissue specimens or by fungal culture or PCR is considered the diagnostic standard in human medicine.^6^ Although veterinary references note the limitations of culture, it is used in certain cases.^7^, ^8^, ^9^ Limitations include unknown diagnostic sensitivity, long turnaround time, and risk to laboratory personnel from exposure to infectious mycelia. In addition, 1 study reported that 81/449 (18.0%) cats and 145/397 (36.5%) dogs from an enzootic area had positive culture results, primarily from tracheobronchial lymph nodes, without finding organisms on histopathology or evidence of clinical disease.^10^ These data bring into question the diagnostic specificity of culture for active disease. Cytopathology has limitations including issues related to the risk associated with sample collection and the possibility of a missed diagnosis if organisms are present in low numbers. Identification of *Histoplasma* yeast organisms in cytology or histopathology samples requires experience because it can appear similar to *Sporothrix*, *Candida*, *Leishmania*, capsule‐deficient *Cryptococcus*, endospores of *Coccidioides*, and small variant *Blastomyces*.^11^, ^12^ In addition, treatment with antifungal drugs can alter yeast morphology, making identification more difficult.^13^ Because of these limitations, serum and urine biomarkers, such as *Histoplasma* antigen, commonly are used to establish a diagnosis in clinically affected cats and dogs.^14^, ^15^, ^16^, ^17^ To determine the clinical utility of fungal culture, a better understanding of its diagnostic performance in combination with cytopathology and antigen detection is needed.

One potential benefit of culture is the ability to evaluate the isolate's susceptibility to antifungal agents. This is important when fungi have unpredictable susceptibility patterns. In human medicine, the susceptibility of *Histoplasma* spp. to commonly used drugs is predictable and therefore testing is not routinely recommended.^18^ Several studies have reported susceptibility patterns, but these are either from clinical isolates obtained from humans or the source was not reported.^19^, ^20^, ^21^ For example, for 23 clinical mycelial isolates from humans the mean minimum inhibitory concentrations (MICs) were 7.0, 0.04, 0.05, and 0.1 μg/mL for fluconazole, itraconazole, posaconazole, and voriconazole, respectively.^22^ Similar patterns have been found from isolates around the world.^22^ Little data is available for clinical isolates from veterinary species. Based on limited genomic data, isolates causing histoplasmosis in cats have substantial genetic divergence from those infecting humans.^23^ Thus, cryptic (only differentiated genetically) species infecting cats or dogs are possible and might have unique susceptibility patterns. A better understanding of the antifungal susceptibility of veterinary isolates is needed to guide diagnostic and treatment decisions in these species.

Our primary aim was to determine the utility of fungal culture when combined with cytopathology and antigen detection for the diagnosis of histoplasmosis in cats and dogs. A secondary aim was to report the antifungal susceptibility patterns of isolates causing histoplasmosis in cats and dogs.

## 2 MATERIALS AND METHODS

### 2.1 Animals

Ours was a prospective cohort study. Client‐owned cats and dogs suspected to have histoplasmosis were recruited from five veterinary referral hospitals. Studies were approved by the respective institutional animal care and use committees. Pet‐owner signed consent was collected before enrollment. Inclusion required a clinical suspicion of histoplasmosis (new diagnosis or relapse) and collection of affected tissue or body fluid. Diagnostic testing included attempted fungal culture of the tissue or fluid sample(s) and cytopathology. Clinical data such as results of *Histoplasma* antigen enzyme immunoassay (EIA; MVista *Histoplasma* Quantitative Antigen EIA, MiraVista Diagnostics, Indianapolis, Indiana) on urine and other clinical information pertinent to the final diagnosis were recorded. Treatment and outcome data were collected for a subset of animals with disease relapse. Treatment decisions were at the discretion of the attending clinician and pet owner.

### 2.2 Clinical diagnosis

The diagnosis of proven histoplasmosis was defined by the identification of *Histoplasma* yeast associated with inflammation on cytopathology. A diagnosis of probable histoplasmosis was defined as findings consistent with ocular or pulmonary involvement (tissues less commonly sampled) and detection of *Histoplasma* antigen in urine by EIA. Evidence of intrathoracic involvement included radiographic changes consistent with parenchymal lung disease or lymphadenopathy. Evidence of ocular involvement included inflammatory disease of the posterior segment, anterior segment, or both. If cytopathology and antigen testing were negative but fungal culture was positive, this situation was defined as proven histoplasmosis as long as additional supportive clinical evidence was present, including pyogranulomatous inflammation on pathology and a positive clinical response to antifungal drug treatment. Relapse was defined as the diagnosis of proven or probable histoplasmosis after discontinuation of all antifungal agents at the recommendation of the attending veterinarian.

Control animals were those that tested negative for histoplasmosis and were classified as either having a definitive alternative diagnosis or unknown diagnosis. Animals with a definitive alternative diagnosis required negative cytopathology for *Histoplasma* organisms along with clinical signs consistent with the alternative diagnosis and specific additional supportive evidence. More specifically, an animal with respiratory disease was required to have cytopathology (lung lavage or lung aspirate) or histopathology (nasal biopsy) supportive of the alternative diagnosis. Cardiac disease required echocardiography. Animals with an alternative diagnosis causing gastrointestinal (GI) disease were required to have supportive findings on GI histopathology. For patients with nonregenerative anemia, a bone marrow aspirate or biopsy was required. For the diagnosis of primary liver disease, an aspirate and cytopathology or liver biopsy and histopathology documenting the alternative diagnosis was required. If joint effusion or lameness was present, synovial fluid cytopathology was required. The diagnosis of cancer required appropriate findings on cytopathology or histopathology. Diagnosis of an infectious disease required cytopathology, histopathology, microbial culture, or other supportive infectious disease testing. An animal with an unknown diagnosis was defined as 1 that did not meet the definition of proven or probable histoplasmosis or an alternative diagnosis.

### 2.3 Fungal culture

Samples suspected to be infected by *Histoplasma* were collected by fine needle aspirate (FNA) for tissues, needle centesis for body fluids, swab for cutaneous or rectal mucosa samples, and peripheral venipuncture for blood. Samples were transferred immediately to separate culturettes for each sample containing either liquid Amies or Stuart media on foam or Amies agar gel (Transystem, Copan Diagnostics, Murrieta, California). Whole blood was transferred immediately to standard aerobic blood culture broth (Bactec Standard Aerobic medium, BD, Franklin Lakes, New Jersey). Samples were submitted to 1 of 2 service laboratories (MiraVista Diagnostics, Indianapolis, Indiana; Oklahoma Animal Disease Diagnostic Laboratory, Stillwater, Oklohoma) with experience in fungal culture. Samples were plated on Sabouraud's dextrose, Mycobiotic, and brain heart infusion with 5% blood agar or potato dextrose and brain heart infusion with 5% blood agar and incubated at 25°C and 37°C in room air atmosphere. If fungal growth was present, identification of *Histoplasma* was based on morphology, 28S rDNA sequencing (Eurofins, Genomics, Louisville, Kentucky), or both. Culture was considered negative if *Histoplasma* was not grown within 6 weeks of plating.

### 2.4 Antifungal susceptibility testing

For a subset of *Histoplasma* culture isolates, antifungal susceptibility testing was performed at a commercial laboratory (Fungal Testing Laboratory, University of Texas Health, San Antonio, Texas). Clinical and Laboratory Standards Institute (CLSI M38‐A2) broth dilution methods were used to determine MICs for fluconazole, itraconazole, posaconazole, voriconazole, terbinafine, and amphotericin‐B. These were reported as μg/mL. For MICs below or above the lowest or highest tested concentration, respectively, that concentration was used for statistical analysis.

### 2.5 Statistical analysis

Statistical analysis was performed using commercial software (SigmaPlot 14.0, SYSTAT Software, San Jose, California). The Shapiro‐Wilk test was used to determine that antifungal susceptibility data was nonparametric (*P* < .001). Descriptive statistics were reported as median and range for continuous variables and frequency and percentage for nominal variables. *Histoplasma* antigen detection by EIA had a quantifiable range of 0.4 to 19 ng/mL. Results above the diagnostic cutoff, but below or above the quantifiable range were considered 0.4 and 19 ng/mL, respectively. Diagnostic sensitivity (true positive/[true positive + false negative]) was calculated for fungal culture, cytopathology, and *Histoplasma* antigen EIA for animals with proven or probable histoplasmosis, with the gold standard being the final clinical diagnosis. Diagnostic specificity (true negative/[true negative + false positive]) was calculated for fungal culture. The McNemar change test was used to compare diagnostic sensitivity among cytopathology, fungal culture, and antigen detection. The sensitivity of the combination of diagnostic testing methods also was determined. Fisher's exact test was used to compare frequency of positive fungal culture between cats and dogs. The Mann‐Whitney *U* test was used to test differences between MICs of fluconazole and voriconazole for isolates from animals with clinical relapse that were previously exposed to fluconazole vs those not previously exposed. The ratio of area under the concentration curve (AUC) from previously published pharmacokinetic studies over the median MIC determined in our study was reported. Statistical significance was set as *P* ≤ .05.

## 3 RESULTS

### 3.1 Cats and dogs with histoplasmosis

Thirty‐three cats with histoplasmosis were enrolled including 30 cats with proven and 3 with probable histoplasmosis. The median age was 3.5 years (range, 0.5‐16.5). Domestic shorthair was the most common breed (n = 22), followed by domestic longhair (7), Siamese (3), and Persian (1). Castrated males (n = 19) were most common followed by spayed females (11), intact females (2), and an intact male (1). Twenty‐nine (87.9%) cats represented new diagnoses, and 4/33 (12.1%) cats relapses. Disease relapse was diagnosed a median duration of 9.5 months (range, 3‐12 months) after discontinuation of fluconazole (n = 3) or itraconazole (1).

Nineteen dogs with histoplasmosis were enrolled including 18 dogs with proven and 1 with probable histoplasmosis. The median age was 3.5 years (range, 1.0‐13.0). Spayed females (n = 8) were most common followed by castrated males (5), intact males (4), and intact females (2). Mixed breed (n = 4) was most common followed by miniature schnauzer (3), Labrador retriever (3), and 1 each of German shepherd dog, great Dane, beagle, Yorkshire terrier, Jack Russell terrier, Alaskan husky, Patterdale terrier, Maltese, and miniature poodle. Fifteen (79.0%) dogs represented new diagnoses, and 4/19 (21.1%) relapses. Disease relapse was diagnosed a median duration of 4 months (range, 3‐36 months) after discontinuation of fluconazole (n = 4).

### 3.2 Cats and dogs without histoplasmosis

Eight cats without histoplasmosis were included; median age was 4 years (range, 1.5‐16.5) with 4 spayed females and 4 castrated males. Domestic shorthair was most common (n = 4) followed by domestic longhair (2), Scottish fold (1), and Ragdoll (1). Alternative diagnoses were made in 6 cats including sporotrichosis (n = 1), neutrophilic cholangitis (1), osteochrondrodysplasia (1), immune‐mediated polyarthritis (1), inflammatory bowel disease (1), and unclassified cardiomyopathy with systemic hyperthyroidism (1). Two cats had unknown diagnoses including a cat with an enteropathy primarily affecting the large bowel and a cat with an antibiotic‐responsive fever and lymphadenopathy. Cytopathology and *Histoplasma* antigen EIA on urine were negative in both cats.

Eleven dogs without histoplasmosis were included; median age was 6.5 years (range, 0.5‐14.0), with 8 spayed females and 3 neutered males. Mixed breed (n = 2) and Siberian husky (2) were most common, with 1 each of Bernese Mountain dog, dachshund, German shepherd dog, Jack Russell terrier, Labrador retriever, standard poodle, and boxer. Alternative diagnoses were made in 10 dogs including invasive mold infection (n = 4), metastatic pulmonary carcinoma (2), osteoarthritis (2), pure red cell aplasia (1), and exocrine pancreatic insufficiency (1). One dog had an unknown diagnosis with acquired portosystemic shunting caused by an undefined hepatopathy.

### 3.3 Cats—Cytopathology, fungal culture, and antigen detection

In 33 cats with histoplasmosis, samples for cytopathology and culture were collected from multiple organs in 19/33 (57.6%) cats and from a single organ in 14/33 (42.4%) cats. *Histoplasma* was found on cytopathology of 1 organ in 24/33 (72.3%) cats, multiple organs in 6/33 (18.2%) cats, and not found in 3/33 (9.0%) cats. Organisms often were found in lymph node (n = 13), followed by spleen (9), liver (6), skin (2), joint fluid (1), and oral cavity (1). Clinical findings in 3 cats with probable histoplasmosis included bilateral chorioretinitis, diffuse structured interstitial lung disease, and hepatosplenomegaly (n = 1); diffuse unstructured interstitial with coalescing alveolar lung disease (1); and, diffuse structured interstitial lung disease and hepatosplenomegaly (1). *Histoplasma* antigen concentrations in urine for these 3 cats were 0.94, 5.83, and 11.3 ng/mL, respectively.

*Histoplasma* spp. were isolated from 17/33 (51.5%) cats, including 17/30 (56.7%) with proven and 0/3 with probable histoplasmosis. Positive cultures came from 8/19 (42.1%) cats with multiple organs sampled and 9/14 (64.3%) cats with a single organ sampled. *Histoplasma* antigen was detected in urine by EIA in 32/33 (97.0%). The median antigen concentration was 6.41 ng/mL (range, 0‐19 ng/mL). *Histoplasma* spp. were isolated from mesenteric lymph node in the 1 cat with no detectable antigen in urine. The diagnostic sensitivity was significantly higher for cytopathology (30/33; 91%; 95% CI, 81%‐100%) and antigen detection (32/33; 97%; 95% CI, 91%‐100%), as compared with culture (17/33; 52%; 95% CI, 34%‐69%; *P* < .001) but was not significantly different between cytopathology and antigen detection (*P* = .48; Table 1).

**TABLE 1:** Diagnostic sensitivity of cytopathology, fungal culture, and antigen detection in urine by enzyme immunoassay in 33 cats with proven or probable histoplasmosis.

Test(s)	Diagnostic sensitivity (95% CI)	
Cytopathology	30/33	91% (81%‐100%)
Culture	17/33	51% (34%‐69%)
Antigen (urine)	32/33	97% (91%‐100%)
Cytopathology + antigen	33/33	100%
Cytopathology + culture	30/33	91% (81%‐100%)
Culture + antigen	33/33	100%

Cats without histoplasmosis (alternate or unknown diagnosis) had samples collected from a single organ in 5/8 (62.5%) and multiple organs in 3/8 (37.5%). *Histoplasma* spp. was not isolated from any sample. *Histoplasma* antigen was detected in urine of 1/7 cats tested, with an antigen concentration below the limit of quantification (<0.4 ng/mL).

### 3.4 Dogs—Cytopathology, fungal culture, and antigen detection

In dogs with histoplasmosis, samples were collected from multiple organs in 10/19 (52.6%) and from a single organ in 9/19 (47.4%). *Histoplasma* was found on cytopathology of 1 organ in 12/19 (63.2%), multiple organs in 6/19 (31.6%), and not found in 1/19 (5.3%). Organisms were found in rectal scrape (n = 7), liver (7), spleen (4), lymph node (4), blood (2), and cavitary effusion (1). The dog with probable histoplasmosis had diffuse unstructured interstitial lung disease and antigen concentration in urine of 1.51 ng/mL.

*Histoplasma* spp. were isolated in 15/19 (78.9%) dogs, including 15/18 (83.3%) with proven and 0/1 with probable histoplasmosis. Positive cultures came from 7/10 dogs with multiple organs sampled and 8/9 with a single organ sampled. *Histoplasma* antigen was detected in urine by EIA in 18/19 (94.7%). The median antigen concentration was 11.31 ng/mL (range, 0‐19 ng/mL). *Histoplasma* was found on cytopathology and isolated from a rectal scrape sample in 1 dog with no detectable antigen in urine. The diagnostic sensitivity was not significantly different between cytopathology (18/19; 95%; 95% CI, 85%‐100%), culture (15/19; 79%; 95% CI, 61%‐97%), or antigen detection (18/19; 95%; 95% CI, 85%‐100%; *P* ≥ .25). The diagnostic sensitivity for fungal culture was significantly higher in dogs (79%; 95% CI, 61%‐97%) as compared with cats (52%; 95% CI, 34%‐69%; *P* = .04; Table 2).

**TABLE 2:** Diagnostic sensitivity of cytopathology, fungal culture, and antigen detection in urine by enzyme immunoassay in 19 dogs with proven or probable histoplasmosis.

Test(s)	Diagnostic sensitivity (95% CI)	
Cytopathology	18/19	95% (85%‐100%)
Culture	15/19	79% (61%‐97%)
Antigen (urine)	18/19	95% (85%‐100%)
Cytopathology + antigen	19/19	100%
Cytopathology + culture	18/19	95% (85%‐100%)
Culture + antigen	19/19	100%

Dogs without histoplasmosis (alternate or unknown diagnosis) had samples collected from a single organ in 8/11 (73%) cases and multiple organs in 3/11 (27%). *Histoplasma* was not isolated from any sample. *Histoplasma* antigen was not detected in urine from any dog.

### 3.5 Antifungal susceptibility of *Histoplasma* spp. isolates

**FIGURE 1:**
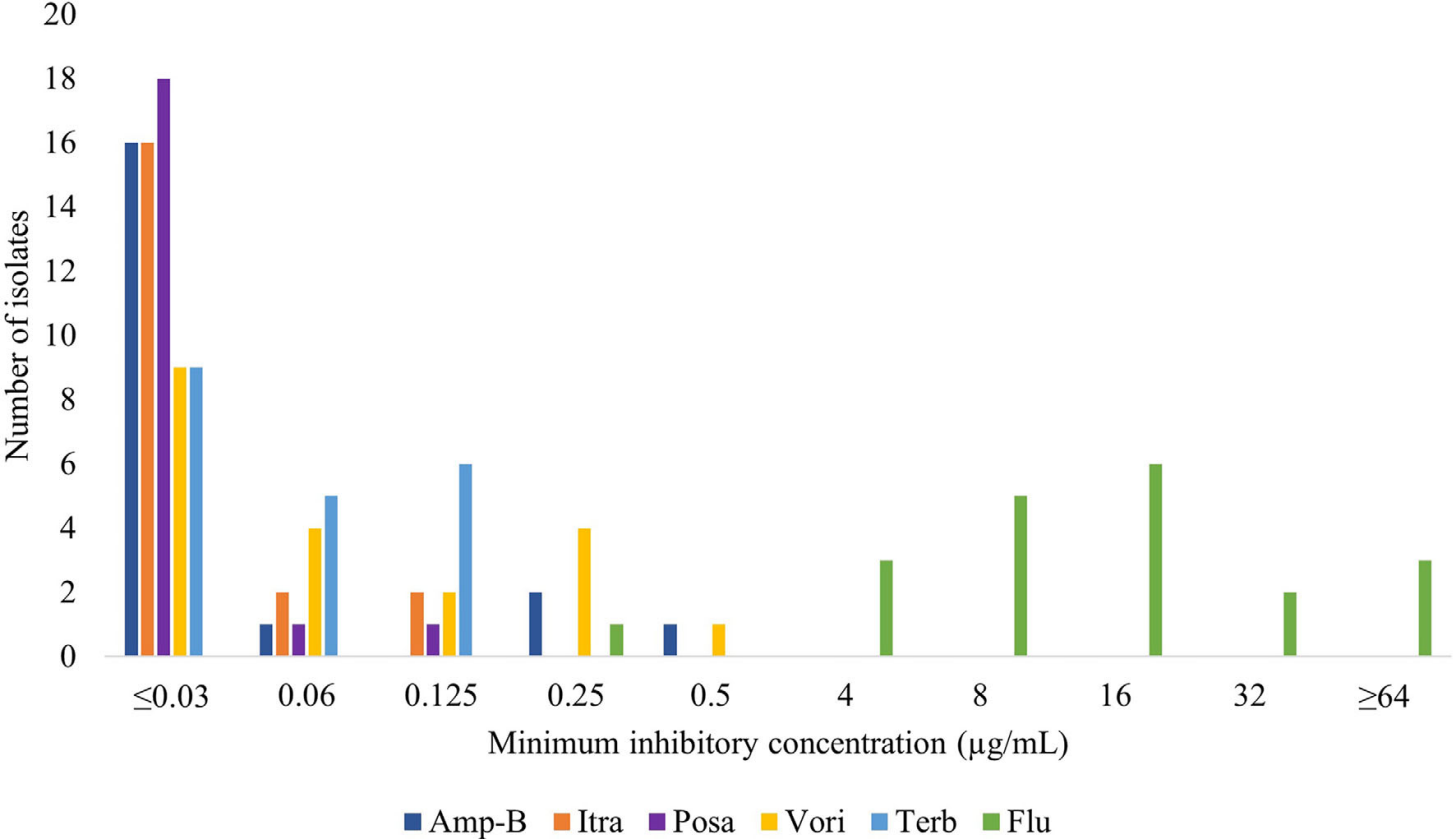
*Histoplasma* spp. isolates from 9 cats and 11 dogs with histoplasmosis and the associated minimum inhibitory concentrations for select antifungal agents. Amp‐B, amphotericin‐b; Flu, fluconazole; Itra, itraconazole; Posa, posaconazole; Terb, terbinafine; Vori, voriconazole.

Susceptibility testing was performed on 9/17 (52.9%) isolates from cats and 11/15 (73.3%) isolates from dogs. Antifungal susceptibility results are shown in Figure 1 and Tables 3 and 4. The median MIC for fluconazole for isolates (n = 5) from relapsed patients that had been previously exposed to fluconazole (48 μg/mL; range, 16 to >64 μg/mL) was significantly higher (*P* = .02) as compared to isolates (15) not previously exposed (11 μg/mL; range, 0.25‐32 μg/mL). The median MIC of voriconazole in relapse isolates (n = 5) previously exposed to fluconazole (0.19 μg/mL; range, 0.125‐0.5 μg/mL) was significantly higher (*P* = .02) than for isolates (15) not previously exposed (0.03 μg/mL; range, <0.03‐0.25 μg/mL). The MICs of relapse isolates to itraconazole were low in all 5 isolates with MICs being ≤0.03 μg/mL (n = 4) or 0.06 μg/mL (1).

**TABLE 3:** Azole susceptibility of *Histoplasma* spp. isolates causing histoplasmosis in 9 cats and previously reported pharmaokinetic data.^a^

	Fluconazole	Itraconazole	Posaconazole	Voriconazole
Median MIC (μg/mL)	16.0	0.03	0.03	0.09
Geometric mean MIC (μg/mL)	10.4	0.04	0.04	0.08
AUC (μg hr/mL; previously reported)	375‐573	15‐70	90	132‐178
Mean oral dose (mg/kg; previously reported)	Tab: 50‐100 mg (total dose)	Sol: 4‐5 Cap: 12.5	Sol: 15	Sol: 4.7 Tab: 4.3
AUC/median MIC	23‐36	500‐2333	3000	1467‐1978
PK reference(s)	[34, 35]	[26]	[52]	[53]

Abbreviations: AUC, area under the curve; Cap, capsule; MIC, minimum inhibitory concentration; PK, pharmacokinetic; Sol, solution; Tab, tablet.

^a^ Data from peer‐reviewed published studies using spectrometry or chromatography with FDA‐approved (human or veterinary species) drug and oral administration. Multidose data used when available.

**TABLE 4:** Azole susceptibility of *Histoplasma* spp. isolates causing histoplasmosis in 11 dogs and previously reported pharmacokinetic data.^a^

	Fluconazole	Itraconazole	Posaconazole	Voriconazole
Median MIC (μg/mL)	8.0	0.03	0.03	0.03
Geometric mean MIC (μg/mL)	12.4	0.04	0.03	0.06
AUC (μg hr/mL; previously reported)^a^	268	9‐28	20‐130	35‐52
Mean oral dose (mg/kg; previously reported)	Tab: 10	Cap: 10‐10.5	Sol: 6 Tab: 7	Tab: 6
AUC/median MIC	33	300‐933	667‐4333	1167‐1733
PK reference(s)	[36]	[27, 28]	[54]	[55, 56]

Abbreviations: AUC, area under the curve; Cap, capsule; MIC, minimum inhibitory concentration; PK, pharmacokinetic; Sol, solution; Tab, tablet.

^a^ Data from peer‐review published studies using spectrometry or chromatography with FDA‐approved (human or veterinary species) drug and oral administration. Multidose data used when available.

Isolates from 5 animals had a MIC to fluconazole ≥32 μg/mL. One *Histoplasma* isolate with a fluconazole MIC of 32 μg/mL was from a newly diagnosed dog that was euthanized without treatment. To our authors' knowledge, this dog did not have prior exposure to azole therapy. Both *Histoplasma* spp. isolates from dogs with MIC >64 μg/mL for fluconazole had relapsed after initial fluconazole treatment. One dog was subsequently treated with a second course of fluconazole at a higher dosage (10.9 mg/kg/day followed by 21.6 mg/kg/day) and achieved clinical remission. The second dog was treated initially and after relapse using compounded fluconazole capsules, (25.5 mg/kg/day, RoadRunner Pharmacy, Phoenix, Arizona) because of pet‐owner financial constraints; this dog ultimately was euthanized because of necropsy‐confirmed progressive disseminated histoplasmosis. A *Histoplasma* spp. isolate from a cat with a fluconazole MIC of 32 μg/mL was from a relapse case initially treated using fluconazole (17.5 mg/kg/day); this cat subsequently achieved a second clinical remission with itraconazole (5.0 mg/kg/day). A *Histoplasm*a isolate from a cat with fluconazole MIC of >64 μg/mL was also from a relapse case initially treated using fluconazole (17.7 mg/kg/day). This cat received a second course of fluconazole (unknown dose) and ultimately was euthanized because of progressive histoplasmosis. Necropsy identified extensive disease, including central nervous system involvement. Because susceptibility testing was performed in batch, results were not available to clinicians making treatment decisions.

## 4 DISCUSSION

Our study showed that fungal culture did not provide any additional benefit when combined with cytopathology and antigen detection for the diagnosis of histoplasmosis in cats and dogs. In addition, antifungal susceptibility patterns were similar to those previously reported from *Histoplasma* clinical isolates obtained from humans.^19^, ^20^, ^21^, ^22^ Susceptibility testing provided evidence of fluconazole resistance, which could be acquired or intrinsic.

The diagnosis of histoplasmosis can be challenging, and several tests might be needed to confirm the diagnosis. Our study investigated the clinical utility of cytopathology, antigen detection in urine, and fungal culture. Culture was not positive in any animal in which cytopathology and antigen detection were negative. Thus, it had no added clinical utility for establishing this diagnosis in our study population. Like the combination of antigen detection and cytopathology, the combination of antigen detection and culture was positive in all animals with histoplasmosis. Because cytopathology has advantages over culture, most notably in turn‐around time and diagnostic sensitivity, clinical scenarios in which fungal culture is needed to establish the diagnosis of histoplasmosis are uncommon.

No animal in our study without proven or probable histoplasmosis had a positive culture. Although inclusion numbers were small, our findings do not corroborate previous reports of apparently healthy animals with positive *Histoplasma* tissue cultures.^10^ There were differences between sample types however as in the previous study samples were collected at necropsy and positive cultures often came from intrathoracic lymph nodes and less often from other lymph nodes, spleen, liver, or adrenal glands. *Histoplasma* spp. are not colonizers or commensals, and a positive culture result from inflamed tissues is considered proof of active disease in humans.^6^ In general, the same interpretation should hold true in veterinary species. The clinical relevance of a positive culture from nondiseased tissue is unclear.

The antifungal activity of azoles is time and concentration‐dependent, whereas that of amphotericin‐B is primarily concentration dependent.^24^ For azoles, this effect is best described using the AUC/MIC ratio. Target AUC/MIC for commonly used azoles, except posaconazole, is >25, and target maximum blood concentration (*C*_max_)/MIC for amphotericin‐B is >10.^24^ The pharmacokinetic parameter that best predicts terbinafine efficacy has not been established, but the duration of time the blood drug concentration exceeds MIC has been used.^25^ The Clinical Laboratory Standards Institute has not established either clinical break points to correlate in vitro susceptibility data to clinical outcomes or epidemiological cut‐offs to differentiate wild‐type from resistant *Histoplasma* strains. Even without these, susceptibility data combined with available pharmacokinetic data can guide treatment decisions. For example, expected blood concentrations of itraconazole at commonly used dosages are approximately 0.5 to 3 μg/mL and AUCs are 17 to 70 μg × hr/mL and 9 to 28 × μg hr/mL in cats and dogs, respectively.^26^, ^27^, ^28^, ^29^, ^30^ This data does not account for the active metabolite, hydroxy‐itraconazole (OH‐itra), which has similar antifungal potency as the parent drug.^31^ In dogs, OH‐itra reaches similar blood concentrations as the parent drug, but concentrations are lower in cats.^30^, ^32^ Even without consideration of this active metabolite, itraconazole should provide adequate exposure based on expected AUC/MIC.^26^, ^27^

Expected blood concentrations of fluconazole, at commonly used dosages (10‐20 mg/kg/day) are approximately 10 to 30 μg/mL in dogs and cats.^33^, ^34^, ^35^ Using AUCs previously reported and the target AUC/MIC of >25, the AUC/ median MIC for fluconazole was 33 in dogs and 23 to 36 in cats. Both values are above the therapeutic target ratio of 25, but only when the higher dose of 100 mg/day is used in cats.^34^, ^35^, ^36^ However, adequate drug exposure would not have occurred for the 5/20 (25%) isolates in our study with MIC ≥32 μg/mL.^34^, ^35^, ^36^ Four of these five isolates were from animals with relapses after previous treatment with fluconazole. These data suggest acquired resistance to fluconazole, which has been described previously in humans after treatment failure.^19^ This conclusion is speculative, because without susceptibility data before treatment, it is not possible to definitively differentiate acquired from intrinsic resistance. Our findings also support the possibility of intrinsic resistance because the *Histoplasma* isolate from 1 dog without prior fluconazole treatment had a high MIC of 32 μg/mL. This dog was not treated and thus clinical response data are unavailable. Collectively, our study findings can be used to help guide clinical decisions and support the use of itraconazole as a first‐line PO treatment for histoplasmosis in cats and dogs, which mirrors current recommendations for humans.^37^ If fluconazole is used, our data suggests dosages at the higher end of the recommended range (20 mg/kg/day) should be used. Using an FDA‐approved product rather than a compounded fluconazole product is advised to ensure consistent potency. Susceptibility testing might be most useful in conjunction with measurement of blood concentrations of fluconazole because of variability in PO bioavailability.^33^, ^38^ Finally, our data support a switch from fluconazole to itraconazole or another triazole if expected clinical improvement is not seen or if disease relapse occurs.

Minimum inhibitory concentrations were consistently low for posaconazole and voriconazole, supporting their use for histoplasmosis in cats and dogs. Based on current drug costs and considering antifungal stewardship, these might be better suited for rescue treatment. Cross resistance between fluconazole and voriconazole has been reported in humans after fluconazole treatment failure.^19^ Significantly higher voriconazole MICs in isolates previously exposed to fluconazole in our study suggest cross resistance might also occur in cats and dogs. These higher MICs however are unlikely to have clinically relevant impact on outcome because the increases in MICs were modest. All isolates had low MICs to amphotericin‐B, and the Cmax/MIC target of >10 should be easily achieved using liposomal or lipid encapsulated amphotericin‐B at standard dosages. Considering that it reaches therapeutic concentrations quickly, our data support the current recommendation of amphotericin‐B treatment for severe or life‐threatening histoplasmosis.^7^, ^8^

All isolates had MICs to terbinafine that were well below expected blood concentrations in dogs. No published data is available on the use of terbinafine to treat histoplasmosis in veterinary species. Reported use of terbinafine for invasive fungal infections in veterinary medicine has been primarily in combination with azoles, where in vitro data indicate synergism against certain organisms.^39^, ^40^ This drug combination, or terbinafine used as a sole agent, has been reported in animals with aspergillosis (systemic or sinonasal and sino‐orbital), various opportunistic invasive mold infections, sporotrichosis, and cryptococcosis.^41^, ^42^, ^43^, ^44^, ^45^, ^46^, ^47^, ^48^, ^49^ Low MICs found in our study support further investigation of terbinafine as a sole agent, or in combination with another class of antifungal drug, for histoplasmosis in cats and dogs.

An antifungal treatment is not usually chosen solely on the basis of antifungal susceptibility. Other factors such as tissue permeation, drug‐to‐drug interactions, tolerability, cost, and availability might influence selection. In our study population, fluconazole was chosen for some animals based on cost and perceived tolerability. Many animals with histoplasmosis can achieve clinical remission with fluconazole monotherapy.^2^, ^17^ Unfortunately, no published data is available from prospective trials designed to compare the efficacy of fluconazole with other triazoles in veterinary species. Retrospective case series have failed to detect significant differences between fluconazole and itraconazole for outcomes such as survival or relapse rate in cats and dogs with histoplasmosis.^2^, ^3^, ^5^ Small patient numbers, incomplete follow‐up, and confounders inherent to retrospective design are important limitations of these studies. Changes in antifungal treatment in animals receiving itraconazole or fluconazole might reflect a lack of clinical response, adverse effects, or both.^2^, ^3^, ^4^, ^50^

Our study had some limitations. Minimum inhibitory concentrations cannot be directly compared among drugs. To provide a context, MIC data was normalized with previously reported drug exposure data (AUC) and a published target ratio (>25) was used for azoles. The clinical relevance of this target remains unknown in cats and dogs. Certain factors not accounted for by this target, such as plasma protein binding (which decreases active drug exposure), anatomic location of infection (body system), and microanatomic location of infection (intracellular vs extracellular) also might affect efficacy. Secondly, clinical disease from *Histoplasma* is caused by the yeast form, but our study used mold for antifungal susceptibility testing. This practice is common because the mold form can be grown more quickly and is thus better suited for diagnostic purposes. Conversion to the yeast form in the laboratory can take up to 8 weeks.^22^ In addition, mold has good agreement with yeast for susceptibility testing.^22^ Differences in MIC can occur between mold and yeast forms for selected isolates however, which could explain a positive clinical response in some animals infected with strains that have high MICs. Higher tissue permeation leading to higher drug concentrations in infected tissues also could play a role. Thirdly, because the inclusion criteria required a sample to be submitted for cytopathology and culture, our study was likely biased against including animals with pulmonary histoplasmosis. In retrospective reviews, this form of histoplasmosis accounts for 18% and 9% of cases in cats and dogs, respectively.^2^, ^5^ Because of risks associated with lung aspiration or anesthesia and bronchoalveolar lavage, many animals with suspected pulmonary histoplasmosis are diagnosed based on clinical signs, thoracic radiographs, and antigen detection. The underrepresentation of pulmonary histoplasmosis in our study could affect external validity. Fourthly, histoplasmosis is a worldwide disease and several *Histoplasma* genomospecies exist, with at least 2 in the United States.^51^ The relatively small number of animals included in our study were naturally infected in 4 US states (Kansas, Oklahoma, Mississippi, and Texas), representing a small portion of the entire world‐wide enzootic area. *Histoplasma* from other geographic regions might have different antifungal susceptibility patterns. Finally, we did not measure blood drug concentrations, and relied on published pharmacokinetic data for interpretation of MIC results. Many pharmacokinetic studies use healthy animals, often of similar age, size, and breed, that are expected to have less variability in drug absorption and metabolism as compared to animals with naturally‐occurring fungal infections. With variable absorption or metabolism, therapeutic drug monitoring is likely required to optimize and individualize dosing.

In conclusion, we found that fungal culture provided no clinical benefit when combined with antigen detection and cytopathology for the diagnosis of histoplasmosis in cats or dogs. Our study provides valuable antifungal susceptibility data using clinical isolates from cats and dogs.

## CONFLICT OF INTEREST DECLARATION

Audrey K. Cook served as Associate Editor for the Journal of Veterinary Internal Medicine. She was not involved in review of this manuscript. Andrew Hanzlicek and Michelle Durkin are employed by MiraVista Diagnostics which offers commercially the antigen detection assay described in this manuscript. No other authors declare a conflict of interest.

## OFF‐LABEL ANTIMICROBIAL DECLARATION

Authors declare no off‐label use of antimicrobials.

## INSTITUTIONAL ANIMAL CARE AND USE COMMITTEE (IACUC) OR OTHER APPROVAL DECLARATION

Approved by Oklahoma State University: VM‐16‐35 (covered OSU, OVS, and KSU), Mississippi State University: 18‐561, and Texas A&M University: 2016‐0318.

## HUMAN ETHICS APPROVAL DECLARATION

Authors declare human ethics approval was not needed for this study.

## References

[1] Sepulveda VE , Williams CL , Goldman WE . Comparison of phylogenetically distinct *Histoplasma* strains reveals evolutionarily divergent virulence strategies. MBio. 2014;5:e01376. 24987093 10.1128/mBio.01376-14 PMC4161242

[2] Wilson AG , KuKanich KS , Hanzlicek AS , et al. Clinical signs, treatment, and prognostic factors for dogs with histoplasmosis. J Am Vet Med Assoc. 2018;252:201‐209. 29319442 10.2460/javma.252.2.201

[3] Reinhart JM , KuKanich KS , Jackson T , et al. Feline histoplasmosis: fluconazole therapy and identification of potential sources of *Histoplasma* species exposure. J Feline Med Surg. 2012;14:841‐848. 22736675 10.1177/1098612X12452494 PMC11108012

[4] Aulakh HK , Aulakh KS , Troy GC . Feline histoplasmosis: a retrospective study of 22 cases (1986‐2009). J Am Anim Hosp Assoc. 2012;48:182‐187. 22474046 10.5326/JAAHA-MS-5758

[5] Ludwig HC , Hanzlicek AS , KuKanich KS , et al. Candidate prognostic indicators in cats with histoplasmosis treated with antifungal therapy. J Feline Med Surg. 2018;20:985‐996. 29256743 10.1177/1098612X17746523 PMC11129244

[6] Donnelly JP , Chen SC , Kauffman CA , et al. Revision and update of the consensus definitions of invasive fungal disease from the European organization for research and treatment of cancer and the mycoses study group education and research consortium. Clin Infect Dis. 2020;71:1367‐1376. 31802125 10.1093/cid/ciz1008 PMC7486838

[7] Sykes J , Taboada J . Histoplasmosis. In: Sykes J , ed. Canine and Feline Infectious Diseases. St. Louis: Elsevier; 2014:587‐598.

[8] Bromel C , Greene CE . Histoplasmosis. In: Greene CE , ed. Infecious Diseases of the Dog and Cat. St. Louis: Elsevier; 2012:614‐621.

[9] Hanzlicek A . Fungal infections. In: Byers CG , Giunti M , eds. Feline Emergency & Critical Care Medicine. Milano, Italy: Edra; 2022:201‐206.

[10] Emmons CW , Rowley DA , Olson BJ , et al. Histoplasmosis; proved occurrence of inapparent infection in dogs, cats and other animals. Am J Hyg. 1955;61:40‐44. 13228411 10.1093/oxfordjournals.aje.a119736

[11] Kauffman CA . Histoplasmosis: a clinical laboratory update. Clin Microbiol Rev. 2007;20:115‐132. 17223625 10.1128/CMR.00027-06 PMC1797635

[12] Guarner J , Brandt ME . Histopathologic diagnosis of fungal infections in the 21st century. Clin Microbiol Rev. 2011;24:247‐280. 21482725 10.1128/CMR.00053-10 PMC3122495

[13] Meinkoth JH , Mitchell C , Cowell R , et al. What is your diagnosis? Cytology of post‐treatment histoplasmosis. Vet Clin Pathol. 1997;26:118‐134.12658588

[14] Cook AK , Cunningham LY , Cowell AK , et al. Clinical evaluation of urine *Histoplasma capsulatum* antigen measurement in cats with suspected disseminated histoplasmosis. J Feline Med Surg. 2012;14:512‐515. 22628269 10.1177/1098612X12450121 PMC11104198

[15] Cunningham L , Cook A , Hanzlicek A , et al. Sensitivity and specificity of *Histoplasma* antigen detection by enzyme immunoassay. J Am Anim Hosp Assoc. 2015;51:306‐310. 26355580 10.5326/JAAHA-MS-6202

[16] Rothenburg L , Hanzlicek AS , Payton ME . A monoclonal antibody‐based urine *Histoplasma* antigen enzyme immunoassay (IMMY[R]) for the diagnosis of histoplasmosis in cats. J Vet Intern Med. 2019;33:603‐610. 30557457 10.1111/jvim.15379 PMC6430878

[17] Clark K , Hanzlicek AS . Evaluation of a novel monoclonal antibody‐based enzyme immunoassay for detection of *Histoplasma* antigen in urine of dogs. J Vet Intern Med. 2020;35:284‐293. 33368653 10.1111/jvim.16006 PMC7848336

[18] Wheat J . Histoplasma. In: Gorbach SL , Bartlett JG , Blacklow NR , eds. Infectious Diseases. 3rd ed. Philadelphia: Lippincott Williams & Wilkins; 2004:2218‐2226.

[19] Wheat LJ , Connolly P , Smedema M , et al. Activity of newer triazoles against *Histoplasma capsulatum* from patients with AIDS who failed fluconazole. J Antimicrob Chemother. 2006;57:1235‐1239. 16627592 10.1093/jac/dkl133

[20] Espinel‐Ingroff A . In vitro antifungal activities of anidulafungin and micafungin, licensed agents and the investigational triazole posaconazole as determined by NCCLS methods for 12,052 fungal isolates: review of the literature. Rev Iberoam Micol. 2003;20:121‐136. 15456349

[21] Espinel‐Ingroff A , Boyle K , Sheehan DJ . In vitro antifungal activities of voriconazole and reference agents as determined by NCCLS methods: review of the literature. Mycopathologia. 2001;150:101‐115. 11469757 10.1023/a:1010954803886

[22] Kathuria S , Singh PK , Meis JF , et al. In vitro antifungal susceptibility profile and correlation of mycelial and yeast forms of molecularly characterized *Histoplasma capsulatum* strains from India. Antimicrob Agents Chemother. 2014;58:5613‐5616. 24982084 10.1128/AAC.02973-14 PMC4135809

[23] Arunmozhi Balajee S , Hurst SF , Chang LS , et al. Multilocus sequence typing of *Histoplasma capsulatum* in formalin‐fixed paraffin‐embedded tissues from cats living in non‐endemic regions reveals a new phylogenetic clade. Med Mycol. 2013;51:345‐351. 23072593 10.3109/13693786.2012.733430

[24] Lewis RE . Current concepts in antifungal pharmacology. Mayo Clin Proc. 2011;86:805‐817. 21803962 10.4065/mcp.2011.0247 PMC3146381

[25] Sakai MR , May ER , Imerman PM , et al. Terbinafine pharmacokinetics after single dose oral administration in the dog. Vet Dermatol. 2011;22:528‐534. 21599768 10.1111/j.1365-3164.2011.00985.x

[26] Mawby DI , Whittemore JC , Fowler LE , et al. Comparison of absorption characteristics of oral reference and compounded itraconazole formulations in healthy cats. J Am Vet Med Assoc. 2018;252:195‐200. 29319446 10.2460/javma.252.2.195

[27] Mawby DI , Whittemore JC , Genger S , et al. Bioequivalence of orally administered generic, compounded, and innovator‐formulated itraconazole in healthy dogs. J Vet Intern Med. 2014;28:72‐77. 24428315 10.1111/jvim.12219 PMC4895533

[28] Hasbach AE , Langlois DK , Rosser EJ Jr , et al. Pharmacokinetics and relative bioavailability of orally administered innovator‐formulated itraconazole capsules and solution in healthy dogs. J Vet Intern Med. 2017;31:1163‐1169. 28627123 10.1111/jvim.14779 PMC5508362

[29] Boothe DM , Herring I , Calvin J , et al. Itraconazole disposition after single oral and intravenous and multiple oral dosing in healthy cats. Am J Vet Res. 1997;58:872‐877. 9256973

[30] Itrafungol [Package Insert]. Fort Worth, TX: Virbac; 2022. https://vet‐us.virbac.com/files/live/sites/virbac‐b2busa/files/client%20leaflet/Products/Itrafungol/20220422%20ITRAFUNGOL_PI.pdf. Accessed January 5, 2023

[31] Odds FC , Bossche HV . Antifungal activity of itraconazole compared with hydroxy‐itraconazole in vitro. J Antimicrob Chemother. 2000;45:371‐373. 10702560 10.1093/jac/45.3.371

[32] Yi Y , Yoon HJ , Kim BO , et al. A mixed polymeric micellar formulation of itraconazole: characteristics, toxicity and pharmacokinetics. J Control Release. 2007;117:59‐67. 17097755 10.1016/j.jconrel.2006.10.001

[33] KuKanich K , KuKanich B , Lin Z , et al. Clinical pharmacokinetics and outcomes of oral fluconazole therapy in dogs and cats with naturally occurring fungal disease. J Vet Pharmacol Ther. 2020;43:547‐556. 32656792 10.1111/jvp.12888

[34] Craig AJ , Ramzan I , Malik R . Pharmacokinetics of fluconazole in cats after intravenous and oral administration. Res Vet Sci. 1994;57:372‐376. 7871259 10.1016/0034-5288(94)90133-3

[35] Vaden SL , Heit MC , Hawkins EC , et al. Fluconazole in cats: pharmacokinetics following intravenous and oral administration and penetration into cerebrospinal fluid, aqueous humour and pulmonary epithelial lining fluid. J Vet Pharmacol Ther. 1997;20:181‐186. 9185083 10.1111/j.1365-2885.1997.tb00093.x

[36] Humphrey MJ , Jevons S , Tarbit MH . Pharmacokinetic evaluation of UK‐49,858, a metabolically stable triazole antifungal drug, in animals and humans. Antimicrob Agents Chemother. 1985;28:648‐653. 3004323 10.1128/aac.28.5.648 PMC176350

[37] Wheat LJ , Freifeld AG , Kleiman MB , et al. Clinical practice guidelines for the management of patients with histoplasmosis: 2007 update by the Infectious Diseases Society of America. Clin Infect Dis. 2007;45:807‐825. 17806045 10.1086/521259

[38] KuKanich K , KuKanich B , Magnin G . Oral fluconazole has variable pharmacokinetics in dogs. J Vet Pharmacol Ther. 2022;46:71‐76. 10.1111/jvp.13101 36300550

[39] Ryder NS , Leitner I . Synergistic interaction of terbinafine with triazoles or amphotericin B against *Aspergillus* species. Med Mycol. 2001;39:91‐95. 11270414 10.1080/mmy.39.1.91.95

[40] Barchiesi F , Falconi Di Francesco L , Scalise G . In vitro activities of terbinafine in combination with fluconazole and itraconazole against isolates of *Candida albicans* with reduced susceptibility to azoles. Antimicrob Agents Chemother. 1997;41:1812‐1814. 9257768 10.1128/aac.41.8.1812 PMC164012

[41] Barrs VR , Halliday C , Martin P , et al. Sinonasal and sino‐orbital aspergillosis in 23 cats: aetiology, clinicopathological features and treatment outcomes. Vet J. 2012;191:58‐64. 21388842 10.1016/j.tvjl.2011.02.009

[42] Stewart J , Bianco D . Treatment of refractory sino‐nasal aspergillosis with posaconazole and terbinafine in 10 dogs. J Small Anim Pract. 2017;58:504‐509. 28485470 10.1111/jsap.12686

[43] Corrigan VK , Legendre AM , Wheat LJ , et al. Treatment of disseminated aspergillosis with posaconazole in 10 dogs. J Vet Intern Med. 2016;30:167‐173. 26566711 10.1111/jvim.13795 PMC4913654

[44] Jaffey JA , Hostnik ET , Hoffman AR , et al. Case Report: Successful management of *Conidiobolus lamprauges* rhinitis in a dog. Front Vet Sci. 2021;8:633695. 33614770 10.3389/fvets.2021.633695 PMC7892434

[45] Crespo‐Szabo SM , Stafford JR . Diagnosis, treatment, and outcome in a dog with systemic *Mycoleptodiscus indicus* infection. J Vet Intern Med. 2021;35:1972‐1976. 34048620 10.1111/jvim.16182 PMC8295661

[46] Troy GC , Panciera DL , Pickett JP , et al. Mixed infection caused by *Lecythophora canina* sp. nov. and *Plectosphaerella cucumerina* in a German shepherd dog. Med Mycol. 2013;51:455‐460. 23294425 10.3109/13693786.2012.754998

[47] Townsell M , Legendre AM , Bemis DA , et al. Long‐term treatment and survival in three apparently immunocompetent dogs with disseminated fungal infection caused by *Phialosimplex caninus* . J Am Anim Hosp Assoc. 2018;54:e54602. 30272483 10.5326/JAAHA-MS-6619

[48] Olsen GL , Deitz KL , Flaherty HA , et al. Use of terbinafine in the treatment protocol of intestinal *Cryptococcus neoformans* in a dog. J Am Anim Hosp Assoc. 2012;48:216‐220. 22474053 10.5326/JAAHA-MS-5813

[49] Viana PG , Figueiredo ABF , Gremiao IDF , et al. Successful treatment of canine sporotrichosis with terbinafine: case reports and literature review. Mycopathologia. 2018;183:471‐478. 29222709 10.1007/s11046-017-0225-6

[50] Hanzlicek AS , Meinkoth JH , Renschler JS , et al. Antigen concentrations as an indicator of clinical remission and disease relapse in cats with histoplasmosis. J Vet Intern Med. 2016;30:1065‐1073. 27158815 10.1111/jvim.13962 PMC5084835

[51] Sepulveda VE , Marquez R , Turissini DA , et al. Genome sequences reveal cryptic speciation in the human pathogen *Histoplasma capsulatum* . MBio. 2017;8:e01139‐17. 10.1128/mBio.01339-17 PMC5717386 29208741

[52] Mawby DI , Whittemore JC , Fowler LE , et al. Posaconazole pharmacokinetics in healthy cats after oral and intravenous administration. J Vet Intern Med. 2016;30:1703‐1707. 27425589 10.1111/jvim.14523 PMC5032877

[53] Vishkautsan P , Papich MG , Thompson GR 3rd , et al. Pharmacokinetics of voriconazole after intravenous and oral administration to healthy cats. Am J Vet Res. 2016;77:931‐939. 27580104 10.2460/ajvr.77.9.931

[54] Kendall J , Papich MG . Posaconazole pharmacokinetics after administration of an intravenous solution, oral suspension, and delayed‐release tablet to dogs. Am J Vet Res. 2015;76:454‐459. 25909378 10.2460/ajvr.76.5.454

[55] Roffey SJ , Cole S , Comby P , et al. The disposition of voriconazole in mouse, rat, rabbit, Guinea pig, dog, and human. Drug Metab Dispos. 2003;31:731‐741. 12756205 10.1124/dmd.31.6.731

[56] Lemetayer JD , Dowling PM , Taylor SM , et al. Pharmacokinetics and distribution of voriconazole in body fluids of dogs after repeated oral dosing. J Vet Pharmacol Ther. 2015;38:451‐456. 25691353 10.1111/jvp.12208

